# Superior Mesenteric Vein Aneurysm With Arteriovenous Communication and Portal Vein Thrombosis: A Rare Presentation in Non-cirrhotic Portal Hypertension

**DOI:** 10.7759/cureus.42595

**Published:** 2023-07-28

**Authors:** Pagadala Nitesh, Pottakkat Biju, Raja Kalayarasan

**Affiliations:** 1 Surgical Gastroenterology, Jawaharlal Institute of Postgraduate Medical Education and Research (JIPMER), Puducherry, IND

**Keywords:** venous aneurysm, superior mesenteric vein aneurysm, portal hypertension

## Abstract

Visceral venous aneurysms are exceedingly rare clinical entities reported in the literature. Venous aneurysms are usually acquired in origin, with most often portal hypertension as the underlying pathology. Most venous aneurysms are incidental findings on imaging. Complications of venous aneurysms like rupture with catastrophic outcomes had been reported. However, no clear guidelines exist regarding the management of portal venous aneurysms as most of the data is available only from case reports. Here, we report a rare finding of fusiform superior mesenteric vein (SMV) aneurysm with arteriovenous communication and portal vein thrombosis in the background of non-cirrhotic portal hypertension.

## Introduction

Visceral venous aneurysms are rare clinical entities reported in the literature. With the portal venous system as the commonest site for visceral venous aneurysm, superior mesenteric vein (SMV) aneurysm accounts for approximately 9% of all visceral venous aneurysms only [1]. Venous aneurysms are usually diagnosed as incidental findings on imaging. Venous aneurysms can be congenital or acquired in origin. Most of the visceral venous aneurysms reported in the literature are acquired in origin, with portal hypertension as the common underlying pathology [1]. Portal hypertension can even result from abnormalities of vascular pattern in the absence of intra- or extrahepatic portal venous obstruction. The other possible acquired causes for venous aneurysms were pancreatitis, cirrhosis, and trauma. The management of portal venous aneurysms is still controversial with current evidence available from very few case reports. Here, we report a rare finding of SMV aneurysm with arteriovenous communication and portal vein thrombosis in a patient with non-cirrhotic portal hypertension.

## Case presentation

**Figure 1:**
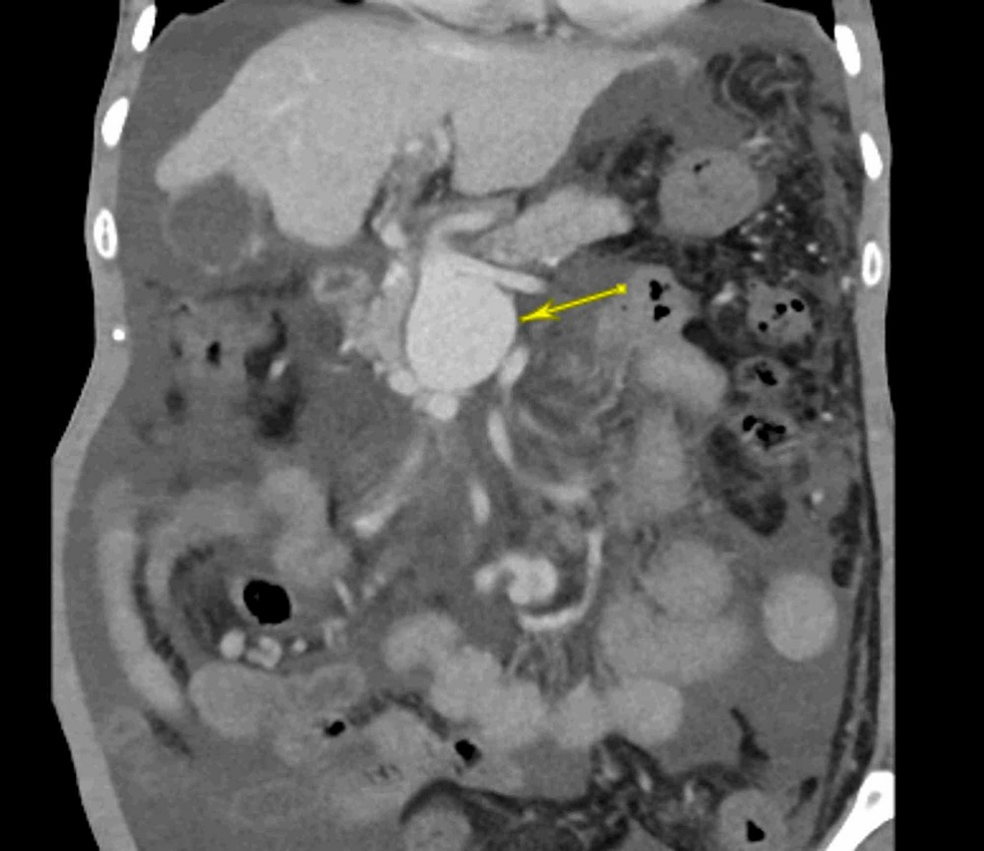
Coronal image of CECT showing fusiform proximal SMV aneurysm (yellow arrow) with preserved splenoportal confluence. Thrombosed portal vein with periportal collaterals and gross ascites can also be noted

A 40-year-old female presented with complaints of abdominal pain in the periumbilical and right upper quadrant regions, which was dull aching in nature and moderate in intensity with no relation to food intake or any aggravating factors for three months duration. She did not have a history of fever, jaundice, gastrointestinal bleeding, or easy fatigability during this presentation. She underwent an open splenectomy for massive splenomegaly approximately 10 years back at another center. She also gave a history of variceal bleeding two years back which was managed with endoscopic variceal banding. She did not have any comorbidity and claims no addictions. On examination, she was thin-built with moderate abdominal distension due to ascites. Her blood investigations revealed a hemoglobin of 10.6 g/dl, a total leucocyte count of 14,660 cells/mm3, and a platelet count of 1,42,000 cells/mm3. Renal function and liver function parameters were within normal limits with serum bilirubin of 0.69 mg/dl, serum albumin of 3.7 g/dl, aspartate aminotransferase of 45 IU/L, alanine aminotransferase of 28 IU/L, and alkaline phosphatase (ALP) of 121 IU/L. Prothrombin time was 14.4 seconds with an international normalized ratio of 0.92. Upper gastrointestinal endoscopy revealed sclerosed esophageal varices with an inflamed fundus. Contrast-enhanced computed tomography (CECT) of the abdomen revealed a focal fusiform aneurysm of the proximal SMV measuring around 4 x 3.5 x 4 cm close to the portal confluence (Figure 1).

**Figure 2:**
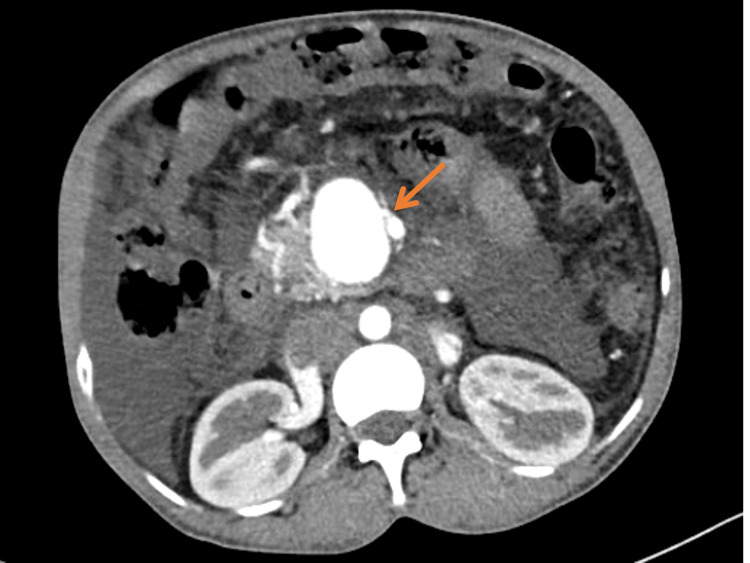
CECT in the arterial phase showing intense arterial enhancement of the SMV aneurysm. Adjacent SMA with arteriovenous communication is also seen (red arrow)

The aneurysm was intensely enhancing in the arterial phase due to arteriovenous communication with the superior mesenteric artery (SMA) (Figure 2).

**Figure 3:**
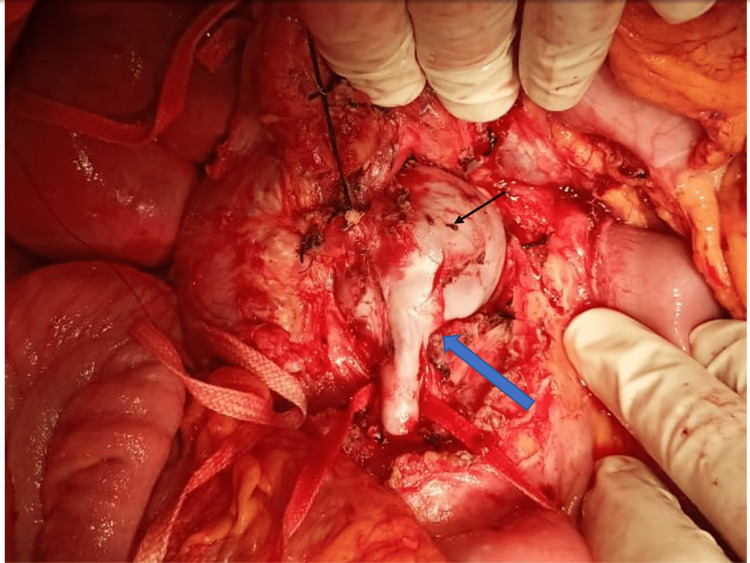
Intraoperative image showing the fusiform aneurysm of the SMV (black arrow) with superior traction on the pancreas neck region. Tapering of the distal end of the SMV was noted (blue arrow)

The main portal vein is thrombosed and replaced by multiple periportal collaterals. The splenic vein was 5.5 mm at the confluence with patent splenoportal confluence. The liver was noted to have irregular margins with no focal lesions. Multiple peribiliary collaterals were noted with central intrahepatic biliary radicle dilation. Multiple mesenteric collaterals and splenic fossa collaterals were noted. Moderate ascites were noted. The symptomatic nature, size, and location of the aneurysm provided irrefutable evidence of the need for intervention. Based on imaging findings of the intense arterial phase enhancement of the aneurysm, with remote suspicion of SMA aneurysm, abdominal aortography was planned initially to further evaluate the aneurysm for possible endoluminal therapy. During abdominal aortography, a large aneurysmal filling in the late phase was noted following contrast injection into SMA, thereby confirming the presence of arteriovenous communication. Due to excessive tortuosity, an attempt to cannulate the aneurysm was futile, and the microcatheter could not be tracked. Therefore, after angioembolization of the aneurysm failed, surgical management of symptomatic aneurysm was considered. Intraoperatively, a nodular liver with massive ascites of approximately three liters was noted. A fusiform aneurysm of proximal SMV measuring 4 X 3 cm was noted. Proximally, SMV aneurysm was noted to be involved till just distal to the splenoportal confluence. Distal narrowing of the aneurysm is noted approximately 2 cm beyond the middle colic vein draining site of the SMV (Figure 3).

**Figure 4:**
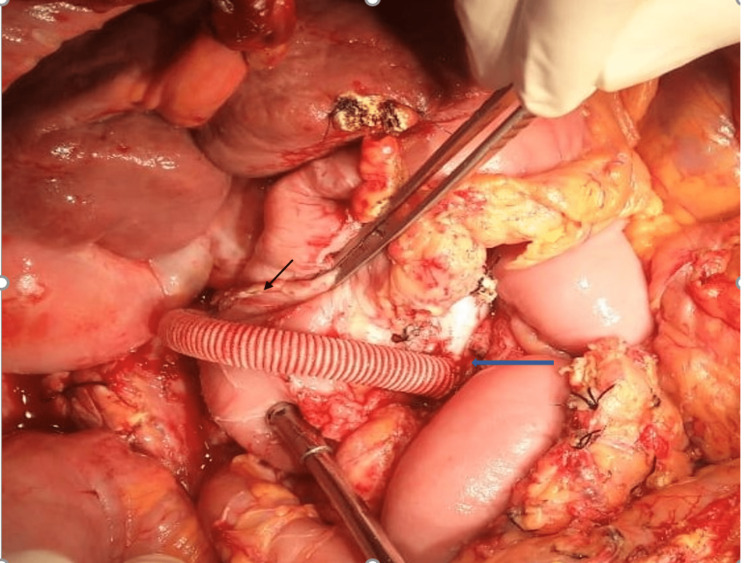
Intraoperative image showing the completed interposition mesocaval shunt over the C-loop of the duodenum (black arrow) and the distal most aspect of the SMV aneurysm (blue arrow)

Dense collaterals were noted in the retroperitoneal region, along the root of the small bowel mesentery and peripancreatic region. The critical location of the SMV aneurysm with the proximal main portal vein thrombosis in the background of portal hypertension ruled out the option of aneurysmectomy. Therefore, an interposition mesocaval shunt was performed with an 8 mm dacron graft using a 6-0 Prolene suture material to minimize the perfusion pressure in the aneurysm, thereby reducing the chances of complications like rupture, thrombosis, and pain (Figure 4).

Her liver biopsy done intraoperatively was reported as non-cirrhotic portal fibrosis. Postoperatively, the patient received an injectable low molecular weight of heparin initially and later titrated with tablet acitrom after 48-72 hours. The patient was discharged after an uneventful postoperative course and on regular follow-up for six months with shunt patency confirmed with a Doppler ultrasound.

## Discussion

In 1982, Schild et al. reported the first case report on an SMV aneurysm in a 60-year-old female during an evaluation for a cystic lesion near the head of the pancreas [2]. Following this, very few reports have been reported in the literature on SMV aneurysms. Aneurysms in the portal venous territory were defined as a dilatation of more than 2 cm in diameter of the vein, especially if morphology is saccular or fusiform [3]. Based on the origin, SMV aneurysms were either congenital or acquired in origin. Non-obliteration of the caudal component of the right vitelline vein all the way down to the caudal-ventral intervitelline anastomosis and forming a small diverticulum is the proposed theory explaining the congenital origin of SMV aneurysms, especially in young patients without any risk factors of portal hypertension. Under the influence of mechanical forces, the small diverticulum enlarges and forms the aneurysm of the proximal SMV. Among the acquired etiologies leading to SMV aneurysms, the presence of portal hypertension with or without underlying liver disease, pancreatitis, and trauma were the predominant etiologies reported. In pancreatitis, the associated peripancreatic inflammatory response, the effect of released proteolytic enzymes on the vessel wall, and the occurrence of portal vein thrombosis can predispose to venous aneurysms. The patient in the present report has a fusiform SMV aneurysm in relation to portal vein thrombosis and a remote history of splenectomy for massive splenomegaly. This explains the acquired factor of portal hypertension as the cause of the SMV aneurysm in the present patient. The SMV aneurysm in the present patient showed intense enhancement during the arterial phase with subtle communication noted with SMA in the CECT abdomen, and a delayed contrast filling of the SMV aneurysm was noted during SMA angiography. All the abovementioned imaging findings corroborate evidence of arteriovenous communication of SMV aneurysm in this patient. The present case report is the first in the literature to highlight the finding of an SMV aneurysm with arteriovenous communication.

Most of the portal venous aneurysms were incidentally detected in asymptomatic patients [4]. Patients with symptomatic aneurysms presented routinely with vague abdominal pain predominantly localized in the right upper quadrant, as was the case with the patient in the present report. The other reported symptoms were upper gastrointestinal bleeding due to either rupture or portal hypertension, jaundice due to compression over the common bile duct, and obstruction related to duodenal compression. The reported complications of SMV aneurysms were thrombosis, rupture, and local compression on adjacent structures [5]. In the largest review on visceral venous aneurysms including 176 patients, the incidence of complete thrombosis was noted in 13.6% of patients, and the incidence of rupture was noted in 2.2% of patients [1]. In those patients with reported rupture, the mean size of the aneurysm was 2 cm only. Even though the reported incidence of the rupture is very low, this finding signifies the unpredictability in the outcomes of SMV aneurysms when stratified based on size criteria alone.

Treatment of SMV aneurysms remains controversial with a spectrum of treatment options varying from close observation to endoluminal therapy and open surgical intervention. In a review by Sfyroeras et al., it was reported that portal venous system aneurysms do not require any form of treatment in most cases. In 88% of patients, who were followed up with abdominal ultrasound alone, the aneurysm diameter remained stable, and no complications occurred in any of the patients [1]. In support of this, there was a report of spontaneous regression of an SMV aneurysm in a patient being followed up for nearly eight years [6]. Only symptomatic aneurysms and aneurysms with the presence of complications like thrombosis, rupture, and compression were considered indications for intervention.

In contrast to the conservative mode of the management of SMV aneurysms, guided only by the evidence of few observations, there has always been a call for surgical intervention even for asymptomatic SMV aneurysms. The proponents for this advocated that the prognosis for these patients with aneurysms is unpredictable and potentially described complications like rupture can be fatal [7]. The lethal potential of rupture developing in a previous asymptomatic SMV aneurysm was reiterated by Smith et al. [8]. In addition, the largest review reported on visceral venous aneurysms included only 8.5% of patients with isolated SMV aneurysms. Hence, observations reported in that review cannot be generalized to all patients with SMV aneurysms. Therefore, an option of surgical intervention must be kept available for all good surgical risk patients with SMV aneurysms. Observative follow-ups should be considered in poor surgical risk patients with stable aneurysms.

The surgical intervention to be chosen depends on the location and shape of the aneurysm, the presence of complications, and associated comorbidities such as liver cirrhosis and portal hypertension. In patients with saccular aneurysms, aneurysmorrhaphy with or without venous patch angioplasty is the best and easiest surgical option preferred. In patients with fusiform aneurysms, aneurysmectomy with interposition bypass graft is the preferred approach. A bypass graft can be either an allograft from a cadaveric donor or a synthetic graft. In patients with documented portal hypertension and liver disease, the aim of surgical intervention is to decompress the portal system through a portosystemic shunt procedure and not to specifically treat the aneurysm. Portosystemic shunting is intended to prevent the ectatic vessel from rupturing and to reverse the dilatation by restoring the pressure in portal circulation to normal. The patient in the present report had the features of portal hypertension with ascites and proximal portal vein thrombosis with multiple collaterals in the periportal, retroperitoneal, and splenic fossa regions. Therefore, the patient underwent an interposition mesocaval shunt to decompress the portal venous system.

## Conclusions

In patients with predisposing conditions, any cystic lesion along the course of superior mesenteric vessels should be evaluated with caution for possible aneurysms. The location, shape, and presence of portal hypertension decide the choice of surgical option in patients with SMV aneurysms. With the minimal evidence available regarding SMV aneurysms and unpredictability in the onset of complications, the option of surgical intervention for SMV aneurysms even in asymptomatic good surgical risk patients cannot be ruled out.
